# Role of Enzymatic Activity in the Biological Cost Associated with the Production of AmpC β-Lactamases in Pseudomonas aeruginosa

**DOI:** 10.1128/spectrum.02700-22

**Published:** 2022-10-10

**Authors:** Isabel M. Barceló, Elena Jordana-Lluch, María Escobar-Salom, Gabriel Torrens, Pablo A. Fraile-Ribot, Gabriel Cabot, Xavier Mulet, Laura Zamorano, Carlos Juan, Antonio Oliver

**Affiliations:** a Research Unit, University Hospital Son Espases-Health Research Institute of the Balearic Islands (IdISBa), Palma, Spain; b Microbiology Department, University Hospital Son Espases, Palma, Spain; c Centro de Investigación Biomédica en Red, Enfermedades Infecciosas, Madrid, Spain; d Department of Molecular Biology, Umeå Centre for Microbial Research (UCMR), Umeå University, Umeå, Sweden; e Laboratory for Molecular Infection Medicine Sweden, Umeå Centre for Microbial Research (UCMR), Umeå University, Umeå, Sweden; Louis Stokes Cleveland VAMC

**Keywords:** *Pseudomonas aeruginosa*, AmpC, ceftolozane-tazobactam, ceftazidime-avibactam, *Galleria mellonella*, *bla*_FOX_ β-lactamases, biological cost, fitness, virulence, peptidoglycan recycling

## Abstract

In the current scenario of growing antibiotic resistance, understanding the interplay between resistance mechanisms and biological costs is crucial for designing therapeutic strategies. In this regard, intrinsic AmpC β-lactamase hyperproduction is probably the most important resistance mechanism of Pseudomonas aeruginosa, proven to entail important biological burdens that attenuate virulence mostly under peptidoglycan recycling alterations. P. aeruginosa can acquire resistance to new β-lactam–β-lactamase inhibitor combinations (ceftazidime-avibactam and ceftolozane-tazobactam) through mutations affecting *ampC* and its regulatory genes, but the impact of these mutations on the associated biological cost and the role that β-lactamase activity plays *per se* in contributing to the above-mentioned virulence attenuation are unknown. The same questions remain unsolved for plasmid-encoded AmpC-type β-lactamases such as FOX enzymes, some of which also provide resistance to new β-lactam–β-lactamase inhibitor combinations. Here, we assessed from different perspectives the effects of changes in the active center and, thus, in the hydrolytic spectrum resistance to inhibitors of AmpC-type β-lactamases on the fitness and virulence of P. aeruginosa, using site-directed mutagenesis; the previously described AmpC variants T96I, G183D, and ΔG229-E247; and, finally, *bla*_FOX-4_ versus *bla*_FOX-8_. Our results indicate the essential role of AmpC activity *per se* in causing the reported full virulence attenuation (in terms of growth, motility, cytotoxicity, and Galleria mellonella larvae killing), although the biological cost of the above-mentioned AmpC-type variants was similar to that of the wild-type enzymes. This suggests that there is not an important biological burden that may limit the selection/spread of these variants, which could progressively compromise the future effectiveness of the above-mentioned drug combinations.

**IMPORTANCE** The growing antibiotic resistance of the top nosocomial pathogen Pseudomonas aeruginosa pushes research to explore new therapeutic strategies, for which the resistance-versus-virulence balance is a promising source of targets. While resistance often entails significant biological costs, little is known about the bases of the virulence attenuations associated with a resistance mechanism as extraordinarily relevant as β-lactamase production. We demonstrate that besides potential energy and cell wall alterations, the enzymatic activity of the P. aeruginosa cephalosporinase AmpC is essential for causing the full attenuation associated with its hyperproduction by affecting different features related to pathogenesis, a fact exploitable from the antivirulence perspective. Less encouraging, we also show that the production of different chromosomal/plasmid-encoded AmpC derivatives conferring resistance to some of the newest antibiotic combinations causes no significantly increased biological burdens, which suggests a free way for the selection/spread of these types of variants, potentially compromising the future effectiveness of these antipseudomonal therapies.

## INTRODUCTION

During the last few years, the balance between antibiotic resistance and associated biological costs has been addressed by several research initiatives as an encouraging source of potential therapeutically exploitable targets (1, 2). This interest is related to the fact that in the current scenario of increasing rates of antibiotic resistance, mainly in top opportunistic pathogens such as Pseudomonas aeruginosa, our therapeutic arsenal is progressively becoming less effective, and as such, new antimicrobial options are urgently needed (3, 4). To minimize this threat, some interesting alternatives are being investigated; for instance, those intended to interfere with bacterial pathogenesis (called antivirulence therapies) in order to increase the probabilities of the clearance of infection by the immune system and, thus, of milder clinical consequences are encouraging but remain at an experimental stage (5). Different strategies in this regard, such as interference with quorum sensing, neutralization of the toxin interaction site, blockade of secretion systems, modulation of the host microbiome, and antiadherence, etc., have been addressed, yet finding targets providing pathogenic attenuation is the essential first step in the development of these kinds of therapies (6). Although some exceptions may exist, and particularities such as the appearance of compensatory mutations, for instance, make the topic very complex, it is generally accepted that resistance is often associated with a fitness cost that impairs virulence, and thus, a deep understanding of the balance between these bacterial features is envisaged as a promising path to identify antivirulence-related Achilles’ heels (1, 2, 7).

Although the interplay between resistance and virulence has been studied from many different perspectives, since the mechanistic pathways enabling resistance are almost endless (1, 2, 7), here, we address the specific issue of β-lactamase production and its associated biological cost. Within this field, some studies demonstrate a lack of significant virulence attenuation associated with the production of different horizontally acquired β-lactamases (8–13). Moreover, the widespread dissemination of several transferable β-lactamases (including extended-spectrum β-lactamases [ESBLs] and carbapenemases) in certain Gram-negative bacteria strongly suggests that their carriage does not represent a significant handicap for fitness/virulence or at least that under antibiotic pressure, many enzymes are positively selected as they provide more advantages than drawbacks for bacteria (14–17). Conversely, other studies show that depending on the species and enzyme considered, the associated biological burden is very important, although the basis for this output is mechanistically variable (8, 12, 13, 18–20). Meanwhile, the topic of the biological cost potentially associated with the mutation-mediated hyperproduction of intrinsic β-lactamases has barely been addressed in Gram-negative bacteria (8). For all of these reasons, the interplay of β-lactamase production and virulence poses a rather intricate topic for which many questions remain unsolved.

Specifically, regarding P. aeruginosa, during the last few years, the interplay between β-lactamase production and virulence attenuation has been studied together with the role of peptidoglycan recycling in this equation. In this sense, the combination of the hyperproduction of the intrinsic AmpC cephalosporinase and peptidoglycan turnover/recycling impairment has been demonstrated to cause dramatic virulence attenuation through different events: impaired growth, the downregulation of the expression of certain key virulence-related genes causing impairments in cytotoxicity and motility, decreased amounts of peptidoglycan per cell, and reduced resistance against immune resources targeting this structure (21, 22). In fact, the peptidoglycan recycling blockade *per se* also partially reduced the virulence of P. aeruginosa in a murine model of infection, pointing toward this process as being a very promising target not only to disable AmpC-mediated resistance but also to dampen pathogenesis (23, 24).

Besides AmpC, the biological burden of expressing different acquired β-lactamases in P. aeruginosa has been shown to be highly variable, and whereas the production of the Ambler class B metallo-β-lactamase VIM-1 or the class A ESBL GES-1 did not have any impact on virulence, the production of various class D OXA-2-type enzymes entailed very interesting differences in the associated virulence attenuations in the Galleria mellonella invertebrate infection model, suggesting that modifications in the active center and, thus, in the hydrolytic behavior of OXA-2-like enzymes have a very important impact on fitness costs (25).

In light of all of these antecedents, knowledge gaps, and the increasing appearance of mutations enabling enzyme variants that provide resistance to new β-lactam–β-lactamase inhibitor combinations such as ceftazidime-avibactam (CAZ-AVI) and ceftolozane-tazobactam (TOL-TAZ) (26–31), which are two of our last resources to combat P. aeruginosa at present, here, we intended to further dissect the biological cost associated with AmpC-type enzyme production. For this purpose, by site-directed mutagenesis, we assessed the role of AmpC β-lactamase activity *per se* in the virulence attenuation associated with its hyperproduction, providing clues to the pathways through which this enzyme is able to dampen bacterial fitness. Moreover, we wanted to ascertain whether several previously described AmpC variants involving P. aeruginosa resistance to CAZ-AVI and TOL-TAZ (T96I [PDC-222], G183D [PDC-322], and ΔG229-E247 [PDC-223]) (32, 33) could carry differential biological burdens compared to the wild-type enzyme, similarly to what happens for OXA-2-like enzymes (25). Finally, we also applied this reasoning to the comparison of two closely related plasmid-encoded AmpC-type β-lactamases, namely, FOX-4 and FOX-8, which display different hydrolytic behaviors, with the former causing resistance to CAZ-AVI and TOL-TAZ and the latter showing a reduced capacity for CAZ hydrolysis compared to its progenitor FOX-3 (34, 35).

This work provides new insights into the AmpC-type β-lactamase-associated biological cost in P. aeruginosa, focusing especially on the role played by the enzymatic activities of these enzymes and variants thereof. Our results suggest that whereas the β-lactamase activity of wild-type AmpC is essential for enabling the above-described full virulence attenuation associated with its hyperproduction under peptidoglycan recycling impairment, no additional important biological burdens seem to be associated with the AmpC-type β-lactamase variants studied. The latter circumstance may ease their selection and spread, posing a worrying threat to the effectiveness of CAZ-AVI and TOL-TAZ as fully reliable antipseudomonal options in the future.

## RESULTS

### Role of wild-type AmpC enzymatic activity in the virulence attenuation associated with its hyperproduction.

**FIG 1:**
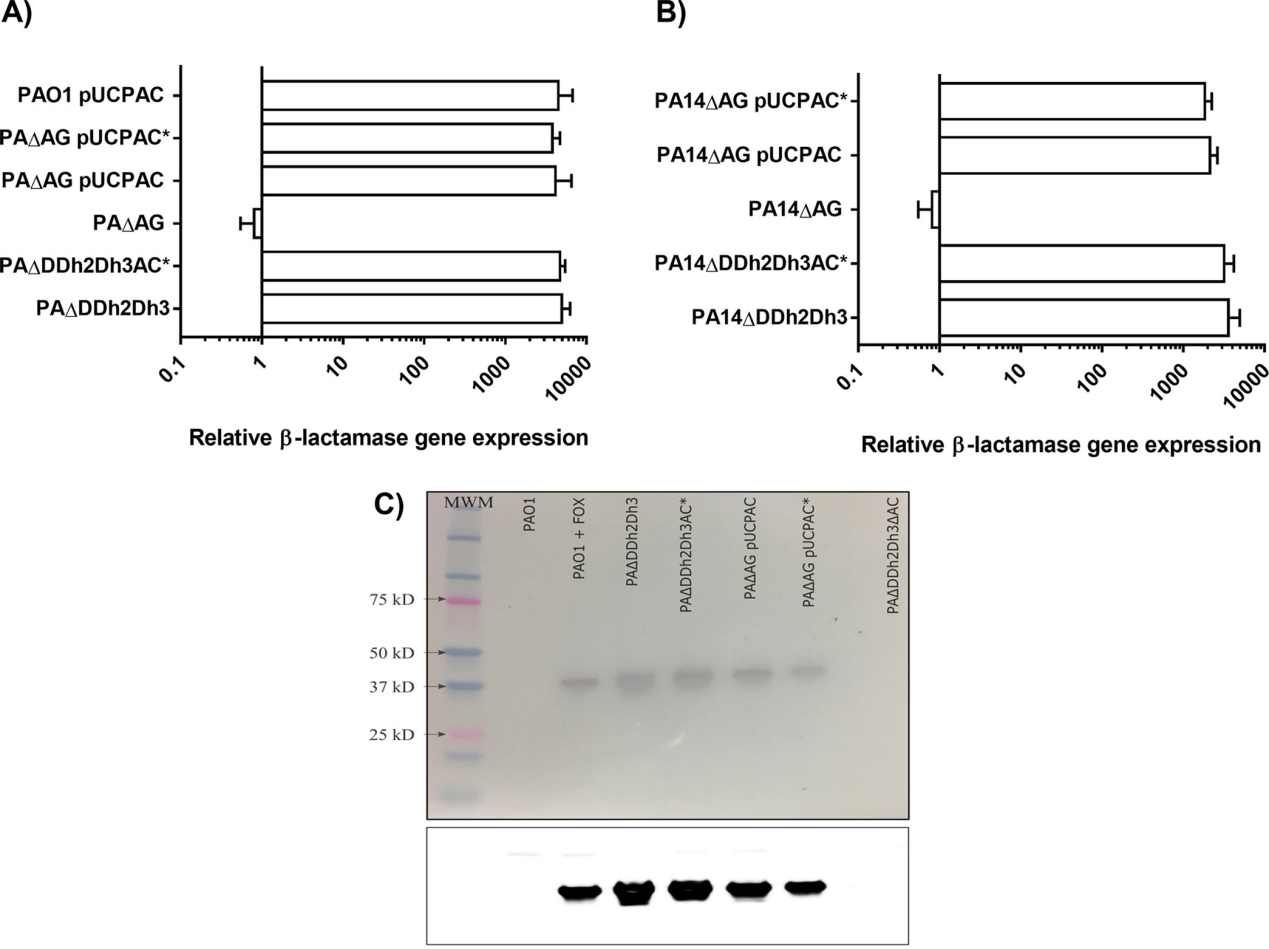
(A and B) Relative (fold) increase/decrease in the mRNA level of each AmpC β-lactamase variant (*ampC* and ampC*) determined by real-time RT-PCR, considering the respective wild-type strain expression level as 1. (A) PAO1; (B) PA14. Horizontal columns represent the mean values from experimental replicates, whereas the error bars correspond to the standard deviations (SDs). All data are displayed on a log scale. The same pair of primers was used to determine the relative expression levels of both *ampC* and ampC* since the site-directed mutagenesis process did not affect the hybridization sequences. (C) Western blot assay with AmpC-specific antibody performed over purified periplasm extracts as explained in Materials and Methods. PAO1 + FOX stands for a culture of PAO1 that was induced with cefoxitin at 50 mg/L (a well-known AmpC inducer) during growth until the OD_600_ reached 0.6. A daylight photograph of the membrane with the molecular weight marker (MWM) (the size of AmpC once the signal peptide is cleaved is ca. 40 kDa) (top) and a chemiluminescence image (bottom) are shown.

To assess the role that the β-lactamase activity of AmpC plays *per se* in obtaining the previously reported virulence attenuation associated with its hyperproduction in peptidoglycan recycling-defective backgrounds (21), we disabled the active center of the enzyme through previously described methods of site-directed mutagenesis (36). More specifically, the essential serine originally present at position 90 of AmpC was replaced by an alanine, providing AmpC* (see Materials and Methods; see also Fig. S1 in the supplemental material). This replacement was performed in both the wild-type PAO1 and PA14 strains and also in triple-amidase-defective backgrounds (PAΔDDh2Dh3 and PA14ΔDDh2Dh3), previously described to be derepressed for AmpC production (since the AmpD-AmpDh2-AmpDh3 amidase homologues also work as indirect *ampC* repressors) and disabled for correct peptidoglycan turnover/recycling (21). Moreover, disabled ampC* was cloned into the pUCP24 multicopy vector and transformed into both wild-type and AmpG permease-defective mutant strains (PAΔAG and PA14ΔAG) in order to obtain another previously described pathway combining hyperproduction and peptidoglycan recycling impairment, also proven to cause significant virulence attenuation (21). To check that the process for disabling β-lactamase activity in ampC*-harboring constructs was correct, their β-lactam resistance profiles and relative *ampC* expression levels were analyzed. As shown in Table 1 and Fig. 1, the production of AmpC* expressed from the mutated gene either in the chromosome or in pUCP24 provided no increase in the MICs of AmpC-hydrolyzable β-lactams. Moreover, the level of hyperexpression of ampC* mRNA was comparable to those of the respective controls (triple-amidase- or AmpG-defective mutants expressing pUCPAC), which indicates that the gene was properly transcribed. To finally ascertain that ampC* was correctly translated and exported, a Western blot analysis was performed with a commercially available antibody (PAO1 AmpC specific) on purified periplasmic extracts. As shown in Fig. 1C, our results suggest that all of the constructs harboring wild-type AmpC or disabled AmpC* accumulated similar amounts of the respective enzymes in their periplasms, which indicates that the correct disabling of AmpC enzymatic activity was achieved without altering the production and location of the protein.

**TABLE 1:** MICs of representative antipseudomonal β-lactams determined in strains harboring wild-type AmpC or its enzymatically disabled variant (AmpC*) or those not expressing the protein because of gene disruption (ΔAC)

Strain	MIC (mg/L)^*a*^							
	CAZ	FEP	P/T	AZT	IMP	MER	TOL-TAZ	CAZ-AVI
PAO1 background								
PAO1	1	1	4	2	1	0.38	0.5	1
PAΔAC	0.75	1	2	2	0.125	0.125	0.38	1
PAO1AC*	0.75	1	3	1.5	0.125	0.094	0.38	0.5
PAΔDDh2Dh3	32	8	>256	24	1.5	1.5	1.5	1.5
PAΔDDh2Dh3ΔAC	0.75	1	2	2	0.19	0.38	0.38	1
PAΔDDh2Dh3AC*	0.5	0.5	1.5	1	0.19	0.38	0.5	0.5
PAΔAG	1	1	4	2	0.5	0.38	0.5	1
PAΔAG/pUCPAC	24	8	>256	12	1	0.75	1.5	1.5
PAΔAG/pUCPAC*	1	1.5	4	2	0.75	0.38	1	1
PAO1/pUCPAC	24	8	>256	16	1	1	1.5	1
PAO1/pUCPAC*	1	1.5	4	2	0.75	0.38	1	1
PA14 background								
PA14	0.75	0.75	3	2	0.38	0.125	0.5	0.75
PA14AC*	0.75	0.75	3	2	0.19	0.125	0.5	1
PA14ΔDDh2Dh3	16	8	>256	32	0.19	0.064	1	1.5
PA14ΔDDh2Dh3ΔAC	0.75	1	3	3	0.19	0.19	0.38	1
PA14ΔDDh2Dh3AC*	0.38	0.5	1.5	1.5	0.125	0.125	0.38	0.75
PA14ΔAG	0.75	1	4	2	0.25	0.19	0.5	1
PA14ΔAG/pUCPAC	12	6	>256	16	0.5	0.38	1	2
PA14ΔAG/pUCPAC*	1	1.5	4	4	0.25	0.125	0.5	1

a CAZ, ceftazidime; FEP, cefepime; P/T, piperacillin-tazobactam; AZT, aztreonam; IMP, imipenem; MER, meropenem; TOL-TAZ, ceftolozane-tazobactam; CAZ-AVI, ceftazidime-avibactam.

**FIG 2:**
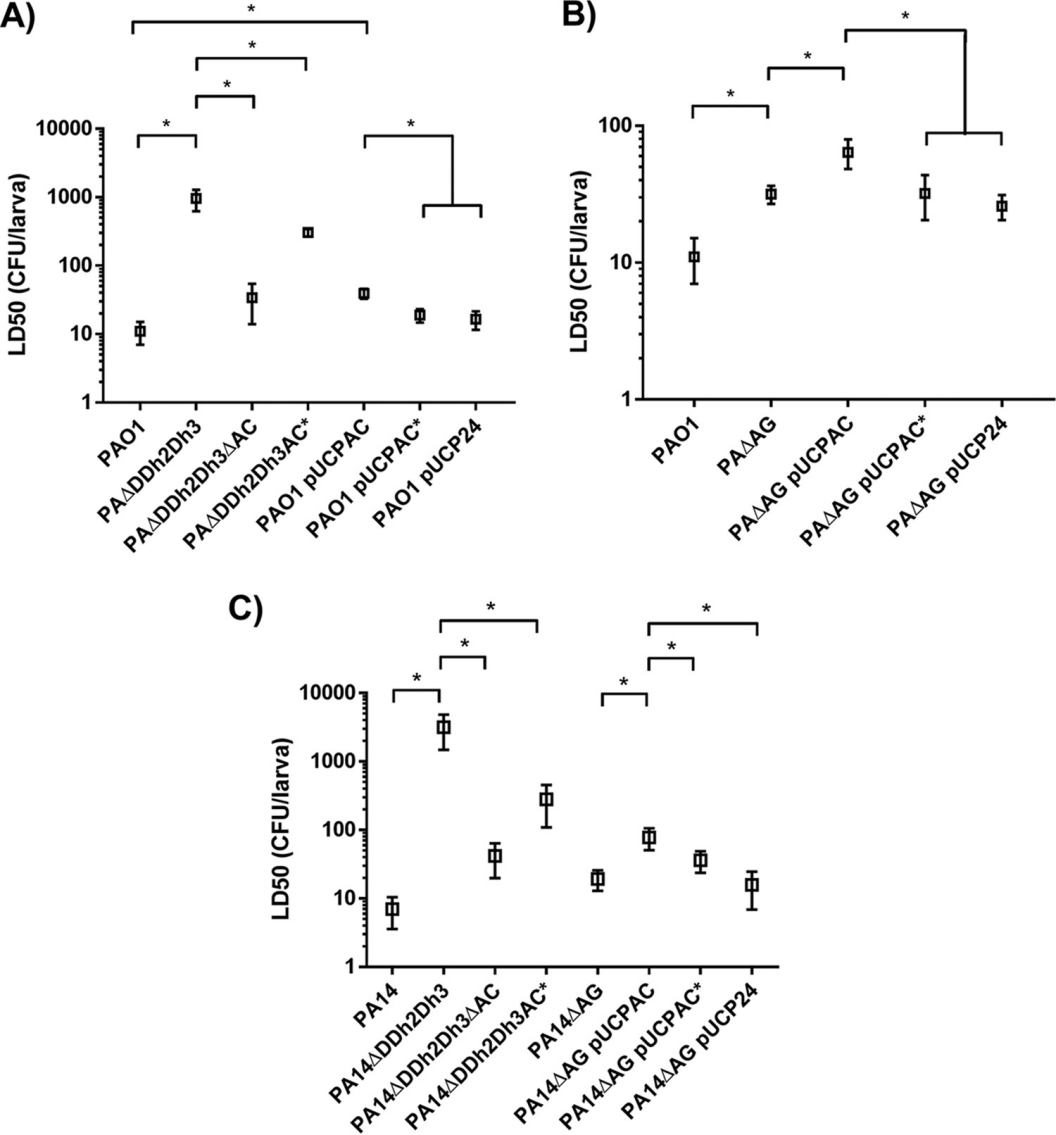
Galleria mellonella killing assays with the different strains expressing AmpC or AmpC* either from the chromosome or from pUCP24-derived constructs. (A) PAΔDDh2Dh3 and PAO1 backgrounds; (B) PAΔAG background; (C) PA14, PA14ΔDDh2Dh3, and PA14ΔAG backgrounds. Mean calculated LD_50_ values, represented by boxes, ± SD (error bars) were obtained from at least three independent experiments. All data are displayed on a log scale. An asterisk over the horizontal bars indicates a *P* value of <0.05 by ANOVA (Tukey’s *post hoc* test) for multiple comparisons between the LD_50_s indicated. Strains are grouped with horizontal lines when there is no statistical difference between them, whereas the symbols for obvious statistical significance are omitted to declutter the figure.

Regarding the G. mellonella killing capacity associated with the production of AmpC versus AmpC*, the results are displayed in Fig. 2. As shown in Fig. 2A, PAΔDDh2Dh3 displayed dramatic virulence attenuation, evidenced through a spectacular increase in its 50% lethal dose (LD_50_), in accordance with previous results (21). Furthermore, accordingly, the disruption of the *ampC* gene and, therefore, the lack of production of the protein were associated with the virtually complete restoration of the wild-type LD_50_ values, indicating that both the peptidoglycan recycling blockade and high-level AmpC hyperproduction were simultaneously needed to enable the full virulence loss reported previously. In other words, the triple-amidase deficiency *per se* or sole AmpC hyperproduction did not cause significant increases in the LD_50_s when occurring separately (Fig. 2A). Interestingly, when the activity of AmpC was disabled in the PAΔDDh2Dh3 background (PAΔDDh2Dh3AC*), the recovery of virulence was notable, with the LD_50_ being ca. 4-fold lower than that of the triple-amidase-defective strain PAΔDDh2Dh3, suggesting that AmpC enzymatic activity is essential (although not sufficient) to cause the full virulence loss reported previously for the triple-amidase-defective strain.

A similar trend, although much less pronounced, was seen when AmpC and AmpC* were produced from their respective genes located in pUCP24-derived plasmids: whereas the expression of pUCPAC in PAO1 entailed a statistically significant approximate duplication of the LD_50_ value, wild-type behavior was restored in the strain expressing pUCPAC*, which showed virtually the same LD_50_ as that of PAO1/pUCP24. As recently demonstrated, the AmpG permease-defective background allows a better appreciation of the biological costs linked to the expression of different β-lactamases (25). In fact, the combination of AmpG disruption (as a paradigm of a peptidoglycan recycling-impaired strain) and AmpC hyperproduction (expressed from the multicopy plasmid pUCPAC) was also previously demonstrated to trigger significant P. aeruginosa virulence attenuation, in clear parallelism with PAΔDDh2Dh3 (21), and for these reasons, we also determined the LD_50_ associated with pUCPAC versus pUCPAC* in PAΔAG. As shown in Fig. 2B, although the absolute LD_50_ values increased in comparison with those in the PAO1 background, the trend of no additional attenuation associated with pUCPAC* compared to the empty vector pUCP24 was conserved, as opposed to the significant increase in the LD_50_ documented for PAΔAG/pUCPAC.

Finally, we wanted to ascertain whether these findings could be translated into another typical P. aeruginosa reference strain, PA14, considered highly virulent and cytotoxic. As shown in Fig. 2C, the same trends as the ones observed for PAO1 were reproduced (although with changes in the absolute numerical LD_50_ values), both when disabling AmpC β-lactamase activity in PA14ΔDDh2Dh3 (PA14ΔDDh2Dh3AC*) and when comparing the expression of pUCPAC to that of pUCPAC* in PA14ΔAG. Thus, altogether, our results suggest that the β-lactamase activity of AmpC notably contributes *per se* to enabling the full biological costs associated with its hyperproduction, although other factors, probably related to energy consumption, also contribute to the attenuated phenotype reported.

### Bases of the differential virulence attenuations associated with the presence versus the absence of functional AmpC activity.

**FIG 3:**
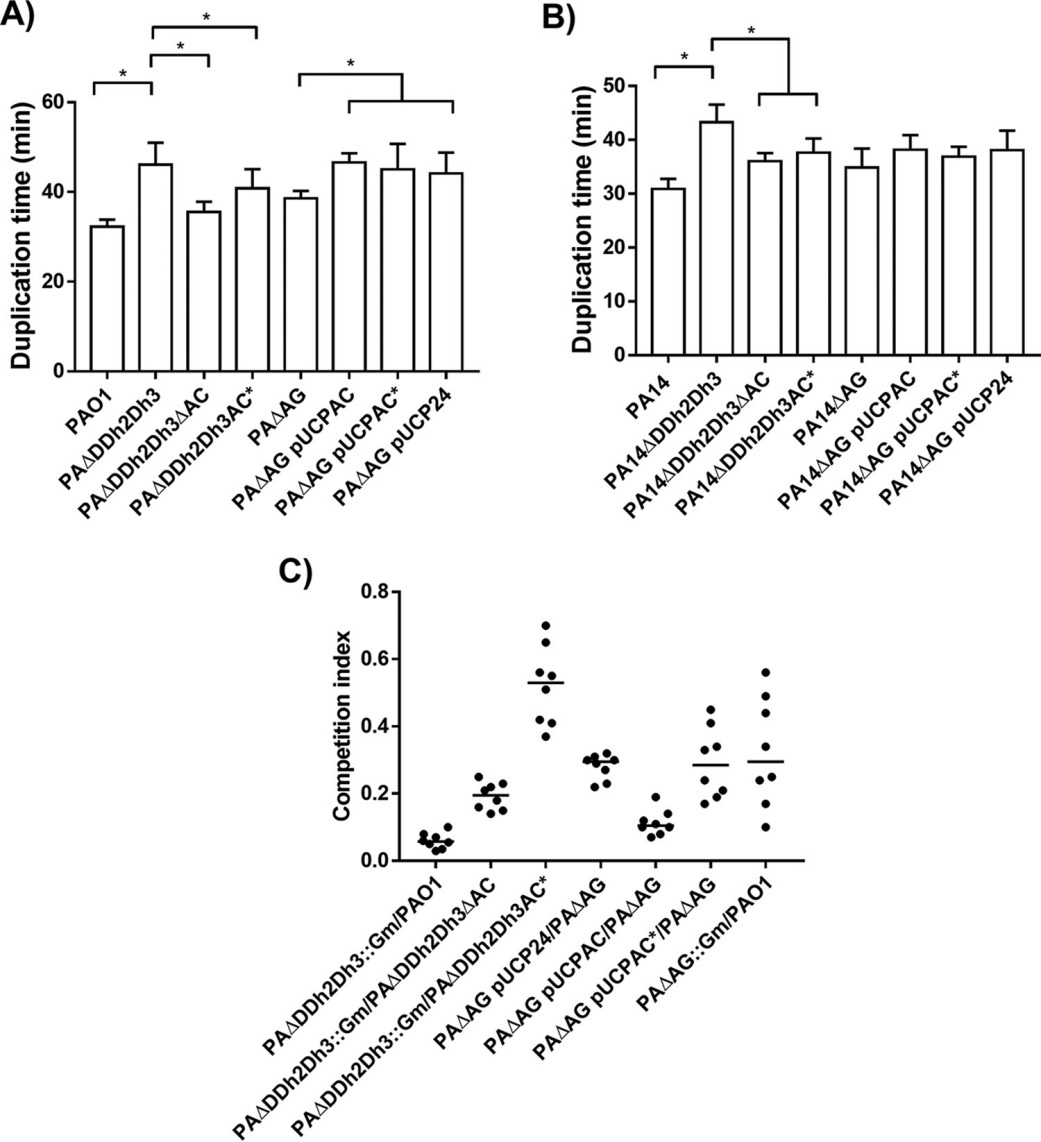
(A and B) Duplication times in minutes of exponentially growing cells of P. aeruginosa PAO1 (A) and PA14 (B) strains and derivatives in LB broth. Values are means plus SD (error bars) from at least three independent experiments. An asterisk over the horizontal bars indicates a *P* value of <0.05 by ANOVA (Tukey’s *post hoc* test) for multiple comparisons between the indicated comparisons. Strains are grouped with horizontal lines when there is no statistical difference between them, whereas the symbols for obvious statistical significance are omitted to declutter the figure. (C) Results of competition experiments. *In vitro* competitions were performed in LB broth flasks in which bacteria were grown at 37°C at 180 rpm for 16 to 18 h, corresponding to approximately 20 generations, as described in Materials and Methods. The CI values obtained for each of the eight independent experiments are plotted. The median CI values are represented by short horizontal bars.

To gain insight into the bases supporting the need for AmpC activity to cause the full attenuation associated with its hyperproduction under peptidoglycan recycling impairment, different fitness- and virulence-associated parameters were characterized, such as growth, competitiveness, cytotoxicity, and motility. As shown in Fig. 3A, and in accordance with previous data (21), the disruption of *ampC* in the PAΔDDh2Dh3 background (PAΔDDh2Dh3ΔAC) caused an almost full recovery of the wild-type duplication time (ca. 35 min), significantly different from that of the triple-amidase mutant (ca. 46 min). Interestingly, when disabling the β-lactamase activity of the enzyme (PAΔDDh2Dh3AC*), although the restoration of the duplication time was not that complete, it was notable and statistically significant (ca. 40 min) (*P* value of <0.05 compared to PAΔDDh2Dh3). Despite not reaching the threshold of statistical significance, similar trends were observed when the duplication time of PAΔAG/pUCPAC was compared to that of PAΔAG/pUCPAC*, with the latter being closer to that of the PAΔAG/pUCP24 control. Similar trends were also obtained for the PA14 strain (Fig. 3B), altogether suggesting that AmpC activity plays a decisive role in impairing the exponential growth of P. aeruginosa under the conditions tested, which would entail an obvious handicap for fitness/virulence.

To assess whether similar effects linked to active versus disabled AmpC could affect the competitiveness of the strains, competition experiments were performed between the strains displayed in Fig. 3C. In accordance with previous results (37), the triple-amidase mutant had a striking reduction in fitness, providing a median competition index (CI) value of less than 0.1 for the PAΔDDh2Dh3::Gm-PAO1 pairing. Interestingly, when *ampC* was disrupted (PAΔDDh2Dh3ΔAC), the competition with PAΔDDh2Dh3::Gm provided quite similar CI values (median value of ca. 0.2), indicating that PAΔDDh2Dh3ΔAC behaved almost like the wild-type strain in this context. Meanwhile, the disabling of AmpC activity meant that the resulting strain did not outcompete PAΔDDh2Dh3 as clearly as PAO1 or PAΔDDh2Dh3ΔAC did, but the PAΔDDh2Dh3::Gm-PAΔDDh2Dh3AC* competition still provided a significant index of 0.53. Similarly, PAΔAG outcompeted PAΔAG/pUCPAC more exaggeratedly than did PAΔAG/pUCPAC* (CI values of ca. 0.1 and 0.3, respectively), indicating a significantly lower fitness cost associated with the production of AmpC* than that for wild-type AmpC. In all cases, the statistical analysis of the distribution of the CI values, performed using the Mann-Whitney U test, provided *P* values of less than 0.05. Altogether, these data suggest that AmpC enzymatic activity notably contributes to the impairment of fitness and, therefore, the competition capacity of strains in which the enzyme is hyperproduced, together with peptidoglycan recycling alterations. Whatever the role that AmpC enzymatic activity plays here, it not only affects exponential growth but also has a notable impact on assays in which the number of generations is much higher, thus reaching a stationary phase, which would pose a clear handicap for virulence.

**FIG 4:**
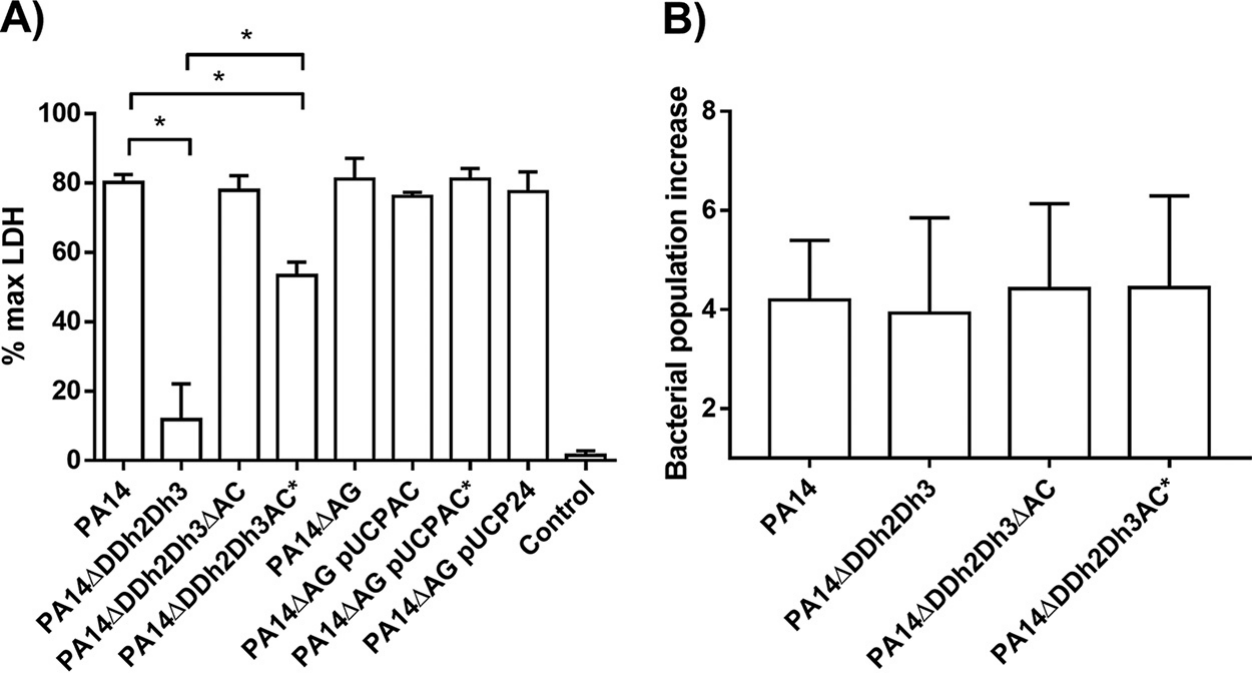
Cell culture infection-related parameters of the strains studied in this work. (A) Cytotoxicity (LDH release) results after infection (MOI of 100 for 3 h) on A549 cells. The results are expressed as a percentage with regard to the maximum LDH that can be released, i.e., from a well of completely lysed confluent cells. The percentage of LDH released by uninfected cells is also shown as a control. (B) Growth of the bacterial population (fold increase) relative to the initial inoculum, under the same conditions as those for cell culture infection in 24-well plates but without A549 cells. For both panels A and B, mean values, represented by boxes, ± SD (error bars) were obtained from at least three independent experiments. An asterisk over the horizontal bars indicates a *P* value of <0.05 by ANOVA (Tukey’s *post hoc* test) for multiple comparisons between the indicated values. The symbols for obvious statistical significance are omitted to declutter the figure.

Figure 4A displays the percentage of maximum lactate dehydrogenase (LDH) released by infected A549 cell cultures, which is used as an indicator of cell death and, thus, the cytotoxic capacity of the different P. aeruginosa strains. As explained in Materials and Methods, PA14 was used in these assays since it is a highly cytotoxic strain that provides interpretable results for this pathogenesis-related feature more easily than PAO1. Whereas the disruption of *ampC* in the triple-amidase-deficient strain (PA14ΔDDh2Dh3ΔAC) entailed the virtually total restoration of cytotoxicity compared to wild-type PA14 (percentage of maximum LDH release of ca. 80%), the disabling of AmpC enzymatic activity (PA14ΔDDh2Dh3AC*) provided lower but still very important recovery, displaying a value of ca. 55% (>5-fold higher than that of PA14ΔDDh2Dh3). Interestingly, although the data described above indicate that PA14ΔDDh2Dh3 displays clear growth defects, the impairment in its cytotoxic capacity was not linked to these since, as shown in Fig. 4B, all of the tested strains behaved in a similar way, i.e., with increases in their populations of ca. 4-fold during infection compared to the initial inocula. Since the conditions here were quite particular (RPMI 1640 medium, 3 h, no agitation, and 5% CO_2_), it was expected that growth would be kept at low rates, therefore entailing minor differences between the strains, as we have demonstrated. All in all, these results indicate that AmpC enzymatic activity not only plays a direct role in impacting the growth of P. aeruginosa when hyperproduced under peptidoglycan recycling alterations but also probably has more specific effects related to the modulation of virulence factor expression, finally affecting the cytotoxic potential regardless of growth.

Curiously, the cytotoxicity of PA14ΔAG/pUCPAC was not significantly altered (Fig. 4A), which is in accordance with the lower degree of attenuation of this strain than PA14ΔDDh2Dh3, in terms of G. mellonella killing (Fig. 2C). This circumstance suggests the existence of particularities in the output and probably the mechanistic basis for the cost linked to each of the two paths used to achieve AmpC hyperproduction and recycling impairment, i.e., triple-amidase deficiency versus AmpG disruption plus pUCPAC expression. In any case, the lack of significant handicaps for the exponential growth and cytotoxicity of PA14ΔAG/pUCPAC compared to the appropriate controls (PA14ΔAG/pUCP24, for instance) is in accordance with previous results regarding the production of pUCP24-cloned OXA-2-like enzymes in the same background, which involved very important alterations in virulence in G. mellonella without affecting cytotoxicity and duplication times (25).

**FIG 5:**
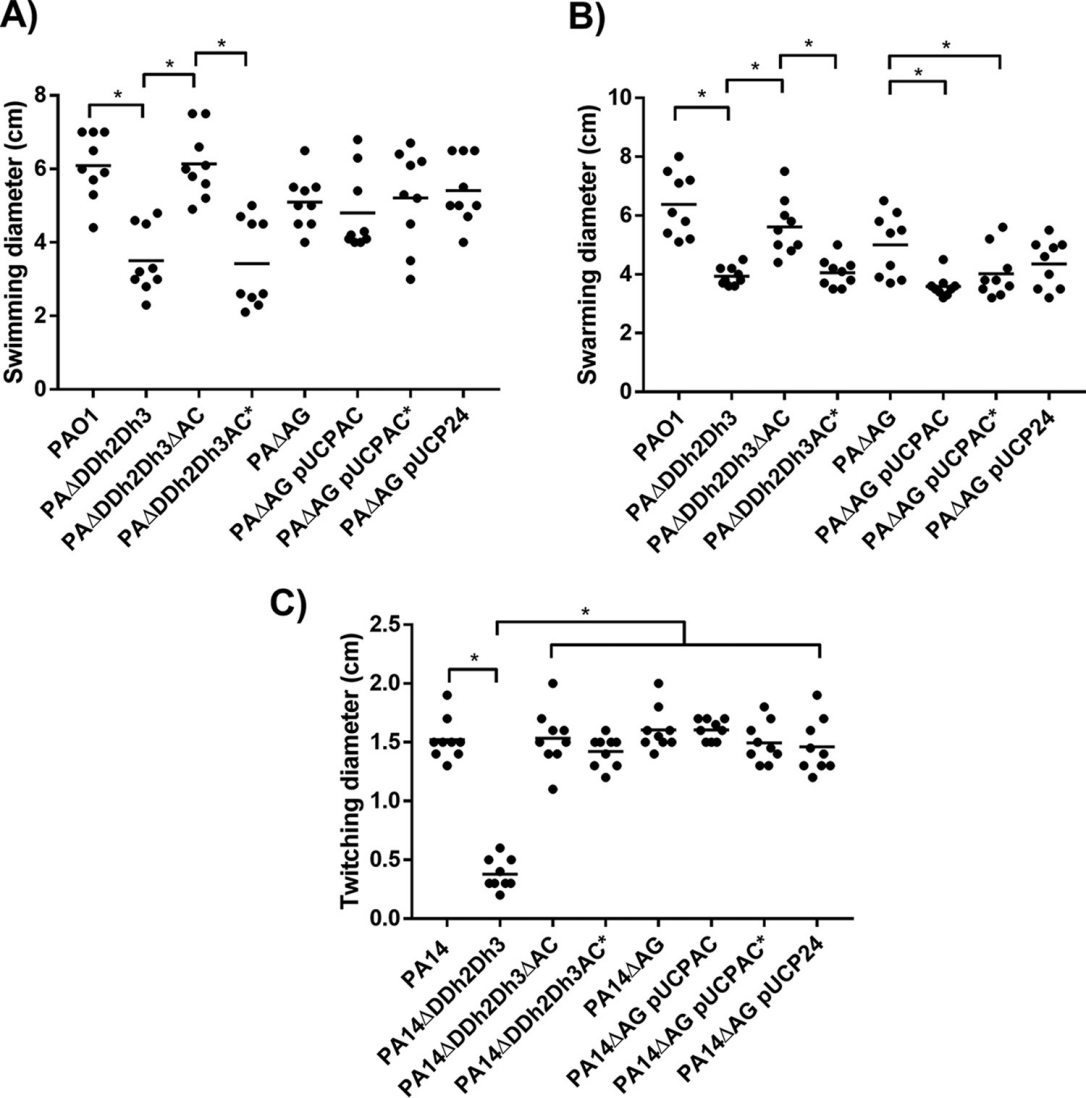
Motility of P. aeruginosa strains studied in this work. The diameters (centimeters) of the motility areas of 9 different inoculations per strain were measured and are depicted by individual symbols. Mean values for the 9 measures are indicated by short black bars. (A) Swimming motility of PAO1 and derived strains. (B) Swarming motility of PAO1 and derived strains. (C) Twitching motility of PA14 and derived strains. An asterisk over the horizontal bars indicates a *P* value of <0.05 by ANOVA (Tukey’s *post hoc* test) for multiple comparisons between the indicated values. Strains are grouped with horizontal lines when there is no statistical difference between them, whereas the symbols for obvious statistical significance are omitted to declutter the figure.

Finally, the different motility types were analyzed as an additional indicator of the fitness/virulence of the strains studied, and the results are shown in Fig. 5. Interestingly, as shown in Fig. 5A and B, whereas the disruption of *ampC* entailed the restoration of wild-type swimming and swarming behaviors, the disabling of enzymatic activity did not have any significant effect on the PAΔDDh2Dh3 phenotype. In other words, the triple-amidase mutant and PAΔDDh2Dh3AC* behaved in the same way, displaying a significant impairment in these two types of flagellum-dependent motility. Therefore, in contrast to the parameters described above, these data suggest that the enzymatic activity of AmpC *per se* does not contribute to the swimming and swarming motility alterations linked to its hyperproduction in the triple-amidase-defective background. In other words, hyperproducing the AmpC* protein, although devoid of activity, could be a significant-enough handicap, energy related in this case, to cause such motility defects. Conversely, the absence of AmpC translation in PAΔDDh2Dh3ΔAC could pose energy savings that are significant enough to enable standard flagellar performance, explaining the wild-type swimming/swarming of this strain.

Regarding twitching motility (Fig. 5C), the disabling of AmpC activity provided results like those for *ampC* disruption, enabling the total restoration of wild-type behavior in the PA14ΔDDh2Dh3 background. These circumstances suggest that in contrast to swimming and swarming, this type IV pilus-dependent motility is affected by AmpC enzymatic activity, at least when hyperproduced in the triple-amidase-defective background. This would pose clear parallelisms with the parameters described above since for lethality in G. mellonella, exponential growth, competitiveness, and cytotoxicity, the disabling of AmpC activity (PAΔDDh2Dh3AC*) entailed important recoveries with regard to wild-type values. However, in the case of twitching, the restoration of wild-type behavior was not only partial but also virtually complete, suggesting an even more intimate linkage between AmpC β-lactamase activity and the regulation of this motility.

Regarding AmpC/AmpC* production from pUCP24-derived plasmids, the motility results are not as clear as those in the triple-amidase-defective background, and although some similar trends can be appreciated, they either did not reach or showed only weak statistical significance. In any case, these results are in accordance with our previous work (25) in which motility was demonstrated to be barely altered in the PAΔAG strain expressing different OXA-2-type enzymes despite the existence of large differences between OXA-2-type enzyme-associated virulence behaviors in G. mellonella.

### Analysis of virulence attenuations associated with the production of different AmpC-type β-lactamase variants.

The P. aeruginosa chromosomal AmpC β-lactamase variants studied in this work were previously described to confer resistance to TOL-TAZ and CAZ-AVI (T96I [PDC-222], G183D [PDC-322], and ΔG229-E247 [PDC-223] [32, 33, 38]). In the case of the plasmid-mediated AmpC-type *bla*_FOX-4_ and *bla*_FOX-8_ enzymes, whereas the former was shown to enable resistance to TOL-TAZ and CAZ-AVI, the latter is a *bla*_FOX-3_ derivative with an impaired β-lactam hydrolysis capacity (34, 35). As shown in Table 2, the antibiograms of engineered strains producing the above-mentioned variants in pUCP24 relative to the parent strains demonstrate the associated phenotypes.

**TABLE 2:** MICs of representative antipseudomonal β-lactams determined in P. aeruginosa strains harboring wild-type AmpC or selected variants and *bla*_FOX-4/8_ enzymes cloned into the pUCP24 plasmid

Strain	MIC (mg/L)^*a*^							
	CAZ	FEP	P/T	AZT	IMP	MER	TOL-TAZ	CAZ-AVI
PAO1	1	1	4	2	1	0.38	0.5	1
PAΔAG	1	1	4	2	0.5	0.38	0.5	1
PAO1/pUCPAC	24	8	>256	16	1	1	1.5	1
PAΔAG/pUCPAC	24	8	>256	12	1	0.75	1.5	1.5
PAΔAG/pUCPAC T96I	32	6	12	8	0.25	0.38	48	12
PAΔAG/pUCPAC G183D	24	4	8	6	0.25	0.5	32	24
PAΔAG/pUCPAC G229-E247	24	3	12	4	0.25	0.38	64	16
PAΔAG/pUCP-FOX-4	>256	24	24	24	0.38	1	24	64
PAΔAG/pUCP-FOX-8	6	3	8	4	0.5	0.38	1	3

a CAZ, ceftazidime; FEP, cefepime; P/T, piperacillin-tazobactam; AZT, aztreonam; IMP, imipenem; MER, meropenem; TOL-TAZ, ceftolozane-tazobactam; CAZ-AVI, ceftazidime-avibactam. MICs for all of the AmpC-type β-lactamase cloned variants in the PAO1 background were the same as those for PAΔAG (not shown).

**FIG 6:**
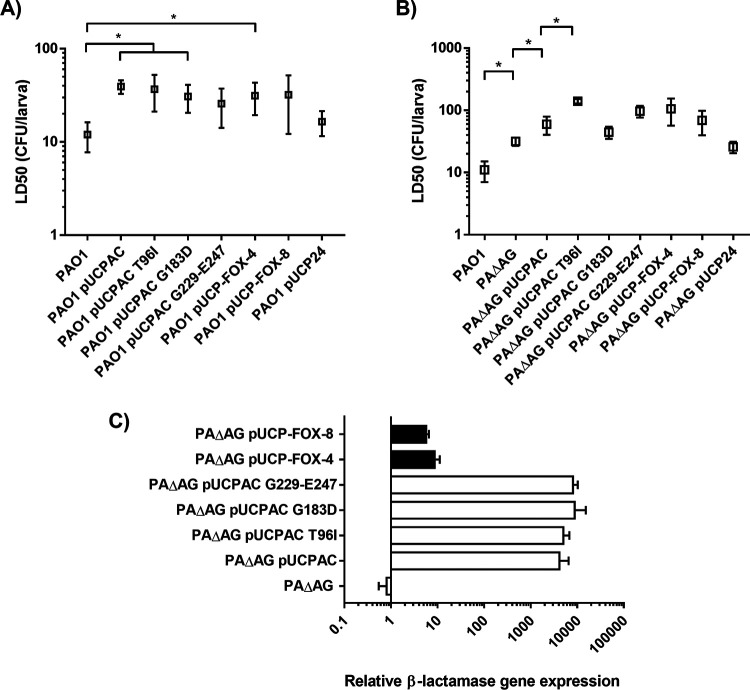
(A and B) Galleria mellonella killing assays with the PAO1 wild-type (A) or AmpG-defective (B) strain expressing the AmpC-type enzyme variants cloned into the pUCP24 plasmid. Mean values, represented by boxes, ± SD (error bars) were obtained from at least three independent experiments. All data are displayed on a log scale. An asterisk over the horizontal bars indicates a *P* value of <0.05 by Tukey’s *post hoc* test for multiple comparisons between the calculated LD_50_s. Strains are grouped with horizontal lines when there is no statistical difference between them, whereas the symbols for obvious statistical significance are omitted to declutter the figure. (C) Relative increase in the mRNA levels of *ampC* or *bla*_FOX_ (black columns) genes cloned into the pUCP24 vector and transformed into PAΔAG, considering the expression level of *ampC* from PAO1 or *bla*_FOX-4_ from the clinical strain HUIGC-PA1 (34), respectively, as 1. Horizontal columns represent mean values from experimental replicates, whereas the error bars correspond to the SD (log scale). The same pair of primers was used to quantify the expression of *bla*_FOX-4_ versus *bla*_FOX-8_ since the hybridization sites do not contain changes for the two genes. The same principle was applied for *ampC* primers since all of the variants showed identical sequences in their hybridization sites.

Regarding the virulence behavior linked to the production of each variant studied here, when determined in the PAO1 background, all of the cloned β-lactamases (including wild-type AmpC) displayed very similar levels of associated attenuation. In other words, the minimal differences in the LD_50_s linked to each variant were never statistically significant between each other but were statistically significant only compared to PAO1, except for the cases of PAO1/pUCPAC G229-E247 and PAO1/pUCP-FOX-8, which, in any case, followed the same trend (Fig. 6A). As mentioned above, PAΔAG is a more suitable background to better appreciate the differences in the biological costs associated with the production of different β-lactamases (25); therefore, accordingly, the G. mellonella killing assays were repeated in this background (Fig. 6B). Although the general trend was similar to that seen for PAO1, some particularities could be observed. For instance, the LD_50_ associated with *bla*_FOX-4_ expression was ca. 2-fold higher than that associated with *bla*_FOX-8_ or wild-type AmpC despite not reaching the threshold for statistical significance. Regarding chromosomal AmpC variants, only the T96I derivative displayed a statistically significant difference in the associated biological cost: its LD_50_ was ca. 2.5-fold higher than that of PAΔAG/pUCPAC, whereas the rest of the variants displayed values very similar to those of the latter strain.

Altogether, these results suggest that no large differences exist regarding the biological costs associated with the production of the AmpC-type enzyme variants enabling TOL-TAZ and CAZ-AVI resistance, compared to wild-type originative β-lactamases. To confirm that the results obtained were reliable and not due to potential differences in the levels of expression of each β-lactamase variant, the relative expression level of each gene was determined compared to appropriate controls, as explained in Materials and Methods. As suggested by the conferred MIC values (Table 2) and as shown in Fig. 6C, no significant differences between the expression levels of different *ampC* or *bla*_FOX_ genes were seen, confirming the validity of our results in the G. mellonella model.

## DISCUSSION

Within the field of the interplay between resistance and fitness/virulence, during the last years, several studies on the balance between the expression of acquired β-lactamases and the associated biological costs were reported, providing a notable variety of results ranging from no significant cost (8–13, 25) to almost unbearable burdens, as happens in Salmonella enterica (8, 12, 13, 18–20, 25). The mechanisms causing these potentially associated biological costs are diverse, including issues related to growth defects, adherence, biofilm formation, and alterations in the peptidoglycan, etc. (12, 13, 18, 19). In the latter regard, the residual dd-endopeptidase activity apparently present in certain β-lactamases, proceeding from a common evolutionary origin with penicillin binding protein (PBP) ancestors, has been proposed as the mechanism that mediates a certain level of peptidoglycan degradation that would finally impair cell wall robustness, resistance against immunity, cell viability, and, consequently, fitness and virulence (8, 13, 18, 22, 25, 39–42). Moreover, some β-lactamases seem closely related to low-molecular-weight PBPs, suggesting that they may sequester/alter the substrates of such enzymes and, thus, interfere with normal peptidoglycan turnover, cross-linking, and final sacculus building/composition, causing negative consequences for its robustness (12).

In any case, besides the acquired enzymes, studies analyzing the biological cost associated with resistance mediated by the hyperproduction of intrinsic β-lactamases are almost nonexistent, although it is a topic that could help unveil therapeutically exploitable antivirulence targets (1, 2, 6, 7, 21). Since the mutation-driven hyperproduction of β-lactamases is a very common resistance mechanism in certain Gram-negative bacteria such as some *Enterobacteriaceae* members or P. aeruginosa, among others (43), it is certainly to be expected that it entails no great handicaps for the bacterium, at least in most situations. However, it has been shown that when high-level AmpC hyperproduction is combined with peptidoglycan turnover/recycling alterations, the fitness and virulence of P. aeruginosa are drastically impaired (21). Through the present work, we wanted to better understand this phenomenon, first seeking to ascertain the role that AmpC enzymatic activity plays *per se* in this context. By using site-directed mutagenesis to disable AmpC activity, we determined that β-lactamase activity is essential but not sufficient to cause the biological cost associated with the combination of peptidoglycan recycling blockade and AmpC hyperproduction. In other words, while disrupting the β-lactamase gene in PAΔDDh2Dh3 virtually reestablished wild-type fitness and virulence, disabling its active center (PAΔDDh2Dh3AC*) only partially achieved this restoration. The most logical explanation for this output is that producing the enzyme (despite being devoid of activity) at thousandfold-higher levels than the wild type in the amidase-deficient and, thus, recycling-altered background may already entail an energy burden that partially impairs fitness although to a lesser extent regarding the attenuation associated with active AmpC. Thus, the basis for the loss of virulence in PAΔDDh2Dh3 and PA14ΔDDh2Dh3 is likely a combination of the energy burden (caused by the alteration of peptidoglycan recycling plus the overproduction of AmpC) and other consequences derived directly from the enzymatic activity of AmpC (discussed below). These results, although less apparent since the initial virulence impairment in terms of the G. mellonella LD_50_ was milder, were corroborated for the other path for achieving AmpC hyperproduction plus peptidoglycan recycling perturbation, namely, PAΔAG or PAΔ14AG/pUCPAC versus pUCPAC* (21).

Delving into the mechanistic bases of these results, the effect of wild-type AmpC activity seems variable depending on the fitness/virulence-related parameters analyzed. Whereas active AmpC was needed to obtain fully impaired exponential growth and competitiveness phenotypes, its impact on swimming and swarming motilities was apparently nonexistent. Thus, producing AmpC or AmpC* in the triple-amidase-defective background had the same effect in impairing these flagellum-dependent motilities, which supports the idea of the energy burden being a key contributor to the attenuated phenotype. In fact, flagellum-dependent motility has been proposed to entail a high energy cost for bacteria (44). Accordingly, from our results, it could be argued that the energy expenditure caused by triple-amidase/peptidoglycan recycling loss combined with AmpC overproduction (regardless of its present/absent enzymatic activity) could be enough to dampen flagellar performance. Conversely, our results indicate that disabling AmpC activity, just like disrupting the *ampC* gene, virtually restored the full capacity for type IV pilus-dependent twitching motility. This was similar for cytotoxicity since the AmpC*-expressing strain recovered ca. 70% of the wild-type cytotoxicity level. These circumstances suggest more than merely energy issues, for instance, some AmpC activity-derived alterations in virulence factor expression, as the cause of impaired twitching/cytotoxicity and, therefore, the attenuated G. mellonella killing capacity in the triple-amidase mutants. In fact, this idea is in full accordance with the previously reported hypoexpression in PAΔDDh2Dh3 and PA14ΔDDh2Dh3 of several genes intimately linked to cytotoxicity (*pcrV*, *pcrH*, *popB*, *popD*, *pscE*, *pscH*, *pscI*, and *exoU-exoS*, all related to the type III secretion system) and the *pilM-pilQ* operon (essential for the biogenesis of type IV pili [21]).

Thus, besides the above-mentioned energy issues, the question that remains without an answer is how AmpC enzymatic activity is able to impact the expression of virulence-related genes in PAΔDDh2Dh3 and PA14ΔDDh2Dh3. One plausible explanation is linked to the above-mentioned conservation of residual peptidoglycan degradation/alteration activity in certain β-lactamases (8, 12, 13, 18, 25, 39–42). This activity would have no appreciable effects under regular conditions but when surpassing certain levels of production, and mostly under peptidoglycan recycling alterations (which in turn would disable an important pipeline of materials for correct cell wall building), could represent an important perturbation for the murein sacculus, entailing (i) cell wall weakening affecting cell viability (8, 13, 18, 22, 25, 39–42), thereby contributing to the exponential growth/competition defects, and/or (ii) a disturbance of the correct *trans*-cell wall assembly of the type III secretion system apparatus or type IV pili, which would obviously dampen virulence (45–47). However, besides these structural aspects, an additional target of this AmpC-reminiscent dd-endopeptidase activity could be not only the sacculus but also the metabolization of derived soluble fragments, known to be continuously released during the processes of remodeling and turnover of peptidoglycan (48). Since peptidoglycan is one of the most dynamic bacterial structures, being highly responsive to external conditions, it is plausible that the released fragments (muropeptides) act as signaling molecules to modulate the bacterial response for better adaptation to each scenario, similarly to what occurs with the regulation of inducible β-lactamases through LysR-type regulators and/or two-component systems (43; M. Escobar-Salom, I. M. Barceló, E. Jordana-Lluch, G. Torrens, A. Oliver, and C. Juan, submitted for publication). In fact, it has been proposed that the differential cytosolic accumulation of certain peptidoglycan fragments can drastically impact the virulence of S. enterica, which, together with some other recently reviewed evidence (49; Escobar-Salom et al., submitted), supports the existence of peptidoglycan fragment-dependent signaling able to modulate bacterial behavior. Thus, we believe that it is not unreasonable to postulate that residual AmpC peptidase activity, when overproduced in a triple-amidase-deficient background, may drive an alteration in the pool of soluble muropeptides and the dependent signaling, causing the modulation of certain genes (such as the above-mentioned genes related to type III secretion and type IV pilus synthesis [21]) and leading to the attenuation of virulence.

This idea could be intimately linked to the fact that the virulence attenuations of PAΔAG and PA14ΔAG/pUCPAC were milder than those of the triple-amidase-defective strains, as shown in Results. Although we considered both paths to be similar ways to achieve high-level AmpC hyperproduction plus peptidoglycan recycling impairment, disrupting the three amidase homologues is obviously not the same as disrupting only AmpG. While this permease is the main gate for the entry of peptidoglycan fragments into the cytosol to be incorporated into recycling reactions, the three amidases have different well-known roles (50–52). Thus, the metabolic effects of disrupting one or the other of these elements must be different, and although the detailed consequences are still unknown, we hypothesize that in PAΔDDh2Dh3 and PA14ΔDDh2Dh3, there is an alteration of presumptive peptidoglycan fragment-related signaling, which, combined with the energy burden and structural impacts on the murein sacculus, would enable the fully attenuated phenotype. Conversely, the likely differential metabolic particularities happening in PAΔAG and PA14ΔAG/pUCPAC would not trigger the above-mentioned alteration of peptidoglycan-dependent signaling. Thereby, peptidoglycan perturbations due to AmpC activity plus the energy expenditure linked to its hyperproduction in the absence of recycling could be sufficient to cause the milder attenuation in these strains.

Obviously, future work will be needed to ascertain all of these hypotheses attempting to link the pathway whereby AmpC enzymatic activity contributes to the phenotypes characterized in this work. Thus, identifying the likely differential qualitative and/or qualitative accumulation of soluble peptidoglycan fragments leading to attenuation in PAΔDDh2Dh3 and PA14ΔDDh2Dh3 (especially affecting the above-mentioned twitching- and cytotoxicity-related genes) as well as the sensors/regulators involved that detect and respond to muropeptide alterations (e.g., LysR-type elements or two-component systems able to bind peptidoglycan fragments [Escobar-Salom et al., submitted]) will be the next step in fully understanding the interplay between AmpC and virulence in P. aeruginosa.

Finally, it is interesting to compare our results with those of other previous studies in which enzymatic activity was not uniformly needed to enable the virulence attenuations associated with certain β-lactamases. Hence, for instance, the catalytic site of the β-lactamase TEM-1 but not that of OXA-3 was required to cause the biofilm formation-deficient phenotypes associated with the production of these enzymes in P. aeruginosa and Escherichia coli (12). Meanwhile, the signal peptide of the protein, but not enzyme activity *per se*, was shown to be intimately linked to the fitness cost associated with the production of SME-1 in E. coli (53). Altogether, these antecedents and our results are suggestive of a very intricate scenario regarding the interplay between β-lactamase production, peptidoglycan metabolism, and virulence, for which general rules valid for all species/enzymes cannot easily be drawn. Thus, future work will be needed to ascertain whether the findings reported here with AmpC and P. aeruginosa could be applicable to other organisms with similar inducible enzymes (43) or even for different horizontally acquired enzymes.

In summary, our work may open new avenues for the development of strategies intended, for instance, to interfere with the above-mentioned peptidoglycan-related signaling or with the correct building of the sacculus, with the final objective of dampening bacterial pathogenesis.

Regarding the last section of our work, the clinical-epidemiological threat posed by increasing rates of antibiotic resistance in P. aeruginosa and other species has been partially alleviated by the introduction of novel β-lactam–β-lactamase inhibitor combinations such as TOL-TAZ, CAZ-AVI, imipenem-relebactam, and others still in clinical development (e.g., meropenem-vaborbactam, meropenem-nacubactam, cefepime-taniborbactam, and cefepime-zidebactam [54, 55]). Although these therapies displayed encouraging levels of effectiveness from the first moment, P. aeruginosa has repeatedly shown the capacity to develop resistance to CAZ-AVI and TOL-TAZ through different mechanisms, posing a real danger to the long-term efficacy of these drugs. In this sense, the acquisition of class B carbapenemases is one of the most threatening mechanisms since only cefepime-taniborbactam retains activity against VIM/NDM-producing P. aeruginosa strains (27, 54, 56). However, there are also some mutation-driven mechanisms, such as the selection of changes in *ampC* and its regulatory genes, that enable the hyperproduction of the enzyme together with an increase in its cephalosporin hydrolysis capacity (obviously including CAZ and TOL) and resistance to inhibition by avibactam. These mutations are usually located in the Ω-loop region of the enzyme or in adjacent residues, which are part of the active site (28). Several mutations in these regions have been reported to confer TOL-TAZ and usually CAZ-AVI resistance, and apart from those studied in this work (32, 33, 38), many other examples exist (27, 30, 32). Similar kinds of mutations selected during treatment, in this case affecting different horizontally acquired enzymes, also enable resistance to TOL-TAZ and CAZ-AVI in P. aeruginosa, for instance, the recently described OXA-2-derived enzyme OXA-681 (57) or the OXA-10-derived enzymes OXA-14, OXA-749, OXA-795, and OXA-824 (31). This would also apply to the plasmid-mediated AmpC-type enzyme FOX-4, which displays high homology with FOX-1, FOX-2, and FOX-3 β-lactamases but with the M11T, A43E, V233A, and Y280H changes providing resistance to TOL-TAZ and CAZ-AVI (35).

Given these antecedents, we sought to assess whether the biological cost associated with the production of this kind of extended-spectrum AmpC-type β-lactamase variants could be significantly different from that of the wild-type originative enzymes. In fact, our previous results regarding different OXA-2-derived extended-spectrum enzymes suggested that changes in the active site of the enzyme had a major impact on virulence (25). We demonstrated that the higher the capacity for cephalosporin hydrolysis of the OXA-2 derivative, the greater the associated virulence attenuation. Thus, we hypothesized that the great biological cost associated with some of the OXA-2-derived ESBLs studied could pose a great handicap for their dissemination (25). Unfortunately, we demonstrate that this phenomenon is not reproduced for the chromosomal AmpC variants studied here or for FOX-4, which, in general terms, displayed biological costs very similar to those of the controls used. It is true, however, that FOX-4 entailed a slight increase in the associated G. mellonella LD_50_ compared to the FOX-8 variant (which does not confer TOL-TAZ or CAZ-AVI resistance), and the same happened for the T96I variant compared to the wild-type AmpC enzyme. However, these increases were only ca. 2-fold, very far from those reported for some OXA-2-type ESBL variants, which were on the order of several hundredfold (25). The particularities of each class of β-lactamase from many points of view (three-dimensional [3D] structure, hydrolytic spectrum, and interactions with substrates, etc. [58–61]) could explain this very different behavior in terms of the impacts that the changes in their active sites and/or surrounding areas have on bacterial virulence. Therefore, general rules assuming that the broader the profile of cephalosporin resistance conferred, the greater the associated fitness cost cannot be deduced for all β-lactamases. Consequently, future work would be needed to ascertain whether our previous findings with OXA-2-like enzymes could be reproducible for other OXA-2- or OXA-10-derived variants conferring resistance to TOL-TAZ and CAZ-AVI (31, 57). The same could be argued for other AmpC variants reported to confer resistance to TOL-TAZ and CAZ-AVI but not yet studied in terms of the associated biological cost (27, 30, 32). Regarding the latter, an interesting idea that must be considered is the fact that the TOL-TAZ and CAZ-AVI resistance appearing in some variants is often associated with a concomitant decrease in the capacity for carbapenem or piperacillin hydrolysis, which could partially support the absence of great increases in the linked fitness cost, in a kind of compensatory-type situation (30, 32, 62).

In summary, our results suggest that the biological cost of the AmpC-type β-lactamase variants studied is comparable to that of the wild-type originative enzymes, which could therefore pose a warning signal: since no additional biological burden seems to be associated with these enzyme derivatives enabling TOL-TAZ and CAZ-AVI resistance that could limit their selection/spread, the progressive appearance of this kind of improved resistance mechanism is likely. Therefore, the judicious use of these new antipseudomonal agents and active surveillance for the appearance and dissemination of such variants are warranted in order to minimize this threat.

## MATERIALS AND METHODS

### Bacterial strains, plasmids, and antibiotic susceptibility testing.

A list and description of the bacterial strains and plasmids used in this work are shown in Table S1 in the supplemental material. Unless otherwise specified, as a general rule, the cloned AmpC-type β-lactamase variants were introduced by electroporation into the wild-type PAO1 or PA14 strain and the respective AmpG-defective mutants (PAΔAG and PA14ΔAG). When indicated for selected strains, susceptibility testing to determine the MIC of representative antipseudomonal β-lactams was performed using MIC test strips (Liofilchem) according to the manufacturer’s instructions and/or Mueller-Hinton broth microdilution according to standard procedures.

### Cloning of the *bla*_FOX-8_ β-lactamase.

To clone the *bla*_FOX-8_ β-lactamase into the pUCP24 multicopy vector, the primers FOX-8-F-EcoRI (TC**GAATTC**ATGCAACAACGACGTGCGTTC) and FOX-8-R-HindIII (TC**AAGCTTT**CACTCGGCCAACTGACTCA) (restriction sites are in boldface type) were used with previously described Escherichia coli clinical strain DNA as the template (35). The PCR products obtained were checked by sequencing, and the resulting plasmid (pUCP-FOX-8) was transformed into E. coli XL1-Blue using the CaCl_2_ heat shock method. After the extraction of plasmids using commercial kits, they were electroporated into the P. aeruginosa strains indicated and then checked again for the absence of mutations by gene sequencing with appropriate primers (Macrogen).

### Analysis of gene expression.

To verify that the cloned AmpC-type β-lactamase variants were expressed at similar levels and, therefore, that potential virulence alterations were not due to different amounts of β-lactamase production, the mRNA of each gene was quantified by real-time reverse transcription-PCR (RT-PCR), according to previously described protocols (21). Total RNA was extracted with the RNeasy minikit (Qiagen) and treated with 2 U of Turbo DNase (Ambion) for 60 min at 37°C to remove contaminating DNA. Next, 5 ng of purified RNA was used for one-step reverse transcription and real-time PCR using the QuantiTect SYBR green RT-PCR kit (Qiagen) in a CFX Connect device (Bio-Rad). The *rpsL* housekeeping gene was used to normalize mRNA levels using previously described primers (21), and the results were referred to PAO1 (to quantify *ampC* variant expression) or a previously described P. aeruginosa clinical strain harboring *bla*_FOX-4_ (HUIGC-PA1 [34]) in order to quantify *bla*_FOX-4_-like gene expression. All RT-PCRs were performed in duplicate, and mean expression levels from three independent RNA extractions were considered. The primers used for these RT-PCRs are listed in Table S2.

### Site-directed mutagenesis to disable AmpC β-lactamase activity.

The AmpC active site is composed of the Ser90 nucleophile within the Ser90-Val-Ser-Lys sequence (63). For its inactivation, a transversion (T→G) at nucleotide 268 was designed, converting the catalytic serine into a structural alanine. *In silico* models of both the native and mutated AmpC were created using Expasy Swiss-model (https://swissmodel.expasy.org/) and compared using the compare tool offered by the website (Fig. S1). Finally, inactivation was confirmed *in silico* using the Provean program (http://provean.jcvi.org/), which determined that the mutation was deleterious, with a score of −3.000.

Site-directed mutagenesis was performed by two-step allelic exchange, according to the protocol described previously by Hmelo et al. (36). The primers used to generate the point mutation are listed in Table S3. Briefly, two different PCRs were designed, both of which included a recognition site for a restriction enzyme on one end and an overlapping sequence containing the point mutation on the other. PCRs were performed using the Platinum *Pfx* high-fidelity polymerase (Invitrogen), according to the manufacturer’s instructions. Finally, a 3rd PCR was performed using the external primers 1F and 3R to concatenate both PCR mixtures containing the desired mutation. The construct was confirmed by Sanger sequencing (Macrogen) prior to cloning into the suicide vector pEX18-Gm using the specified restriction sites. The plasmid pEX18-AmpC* was transformed into P. aeruginosa by conjugation, using E. coli S17-1 as the donor strain with gentamicin as a selective marker. The second allelic exchange prompting vector excision was achieved by growing selected mutants in LB agar containing 50% sucrose at 30°C. Mutants were screened by colony PCR and further confirmed by Sanger sequencing.

### Periplasm content purification and AmpC detection by Western blotting.

The periplasm of the indicated P. aeruginosa strains was fractioned according to a protocol based on spheroplasting by lysozyme and sucrose treatments described previously by Imperi et al. (64), with the following modifications. One liter of culture at an optical density at 600 nm (OD_600_) of 0.6 was centrifuged in a benchtop centrifuge at 3,220 × *g* for 45 min at 4°C. The pellet was washed twice in the washing buffer described above, under the same conditions. The pellet was then resuspended in 250 μL of the spheroplast buffer and incubated for 1 h in a water bath at 70 rpm. After 2 min of incubation, 100 μL of 0.1 M MgCl_2_ was added, and the periplasm fraction was recovered by centrifugation at 3,220 × *g* for 45 min at 4°C. The protein concentration was determined by the Bradford assay (65) and ranged from 1 to 1.7 mg/mL. A total of 1 mg/mL of protein was loaded onto a two-dimensional (2D) protein gel (Bio-Rad) and transferred to a nitrocellulose membrane. The AmpC protein from the different strains was detected using a rabbit anti-PAO1 AmpC primary antibody (Abyntek) and a secondary horseradish peroxidase (HRP)-conjugated anti-rabbit antibody (Invitrogen). The Western blot was revealed using the SuperSignal West Pico Plus chemiluminescent substrate (Thermo Scientific).

### Invertebrate infection model.

The wax moth Galleria mellonella was used as the infection model according to previously described protocols (21, 25). Exponentially growing cultures of the corresponding strains were pelleted, washed, and resuspended in Dulbecco’s phosphate-buffered saline (PBS) without calcium/magnesium (Biowest). Different serial dilutions (depending on the strain) were made in PBS and injected using Hamilton syringes (10-μL aliquots) into individual research-grade G. mellonella larvae (~2-cm-long TruLarv caterpillars; Biosystems Technology) via the hindmost left proleg. Ten larvae were injected for each dilution and strain and scored as live or dead after 20 h at 37°C. An approximate 50% lethal dose (LD_50_) was initially determined in a pilot screen of wide bacterial load intervals (logarithmic scale). When an approximate LD_50_ was obtained, three final experiments with already adjusted bacterial loads were performed, with the final numbers of injected bacteria being verified by plating serial dilutions and colony counts. In all cases, 10 larvae were inoculated with 10 μL of PBS as controls. The percentage of larvae that died at each bacterial dose was modeled and analyzed by Probit analysis, and the LD_50_ ± standard deviation (SD) was finally determined using R software, version 3.2.2 (21, 25). At least three independent LD_50_s per strain were calculated with this model, and a final mean value ± SD was obtained and statistically analyzed as explained below.

### *In vitro* exponential growth rates.

*In vitro* exponential growth rate assays were performed according to standard procedures (21, 25). Briefly, 1-mL samples taken from the corresponding liquid LB cultures grown overnight were diluted in 50 mL of fresh LB broth in 250-mL flasks and incubated at 37°C with agitation at 180 rpm. The doubling times of exponentially growing cells were determined by plating serial dilutions onto LB agar plates at 1-h intervals. At least three independent experiments were performed for each of the selected strains.

### *In vitro* competition experiments.

Pairwise competition assays between the indicated strains were performed according to previously described protocols (37). Depending on the pair of strains analyzed, the resistance marker (gentamicin resistance cassette) was present in the PAΔDDh2Dh3 or PAΔAG strain (PAΔDDh2Dh3::Gm and PAΔAG::Gm); alternatively, the presence of pUCP24-derived plasmids was used as a selector (see Results). To perform the competitions, exponentially growing cells (LB broth) of the corresponding strains were mixed in a 1:1 ratio and diluted in a 0.9% saline solution. Approximately 10^3^ cells from each of the mixtures were inoculated into eight 10-mL LB broth flasks and grown at 37°C at 180 rpm for 16 to 18 h, corresponding to approximately 20 generations. Serial 10-fold dilutions were plated in duplicate onto LB agar and LB agar complemented with 15 μg/mL of gentamicin (LBA-Gm) in order to determine the total CFU and the CFU of the strains with a gentamicin resistance marker, respectively. The competition index (CI) was defined as the gentamicin-resistant strain/gentamicin-susceptible strain ratio. CI values were calculated for each of the eight independent competitions, and the median values were recorded. During the standardization of the procedure, experiments with no mixtures but with only a pUCP24-derived plasmid-harboring strain showed comparable numbers of colonies in LB agar and LBA-Gm plates, ruling out any significant effect of plasmid loss on the final calculated CIs.

### Motility assays.

Swimming, swarming, and twitching motilities were determined for the selected strains, as described previously (21, 25), in plates containing different media. (i) To determine swimming motility, 10 g/L tryptone, 5 g/L NaCl, and 0.3% (wt/vol) mid-resolution agarose were used. Plates were inoculated with an isolated colony from a culture grown overnight in LB agar at 37°C using a sterile toothpick. (ii) To determine swarming motility, 1× M8 minimal medium was supplemented with 1 mM MgSO_4_, 0.2% glucose, 0.5% Bacto Casamino Acids, and 0.5% agar. Aliquots (2.5 μL) were taken from cultures grown overnight to inoculate the surface of the plate. (iii) To determine twitching motility, isolated colonies were inoculated with a sterile toothpick inserted into the bottom of the LB agar plates. In all cases, the plates were wrapped with film to prevent dehydration and incubated at 37°C for 16 h. After incubation, the diameter of the motility zone was measured. For the plates used to determine twitching motility, the medium was taken off the plate, and the print over the bottom was measured. If the motility halo was irregular, two perpendicular diameters were measured, and the result was expressed as the mean. The PAO1 strain and derivatives were used in the swimming and swarming assays, whereas PA14 and derivatives were used for twitching assays since our laboratory collection PAO1 strain is defective in this type of motility. At least 10 determinations for each strain and motility type were recorded.

### Cell culture experiments.

The A549 line of human alveolar type II pneumocytes (Sigma-Aldrich) was used between passages 3 and 30. A549 cells were maintained in RPMI 1640 medium (without phenol red) supplemented with 2 mM glutamine, 10 mM HEPES, 10% heat-inactivated fetal bovine serum, and 1× antibiotic-antimycotic solution (Biowest) at 37°C with 5% CO_2_ and 100% relative humidity. The day before the assays, the cells were seeded at ca. 0.5 × 10^5^ cells per well in 24-well plates. The next day, the cells were ~90% confluent (~1 × 10^5^ cells/well) and were infected with the indicated strains (see Results) at a multiplicity of infection (MOI) of 100, according to previously described protocols (21, 25). Briefly, bacteria from liquid LB broth cultures grown overnight were centrifuged and washed with PBS. Bacteria were then diluted in the above-described RPMI 1640 medium without serum/antibiotic-antimycotic solution (called infection medium [IM]): ca. 1 × 10^7^ bacteria/500 μL of IM were used to infect each well after discarding the initial cell culture medium and washing with PBS. After inoculating the wells with the IM, the plates were centrifuged (200 × *g* for 10 min) to synchronize the arrival of bacteria to the cell monolayer. After an infection period of 3 h, the IM was collected and stored at −80°C to assess the cytotoxicity caused in A549 cells. For this purpose, samples were processed with the cytotoxicity detection kit plus (Roche), which measures lactate dehydrogenase (LDH) release as a marker of cell death, according to the manufacturer’s instructions. To determine the impact of bacterial growth on the cytotoxic capacity of each strain, cell culture infection conditions were reproduced (medium, time, and conditions of incubation) in 24-well plates but without the A549 cell monolayer. Bacterial suspensions were serially diluted and plated at the beginning and end of the incubation to determine the relative increase in the number of bacteria. All of these cell culture-related determinations were always performed with samples proceeding from at least nine wells (three wells from each of three independent plates) per strain.

### Statistical analysis.

With the exception of LD_50_s (see above), GraphPad Prism 7 software was used for statistical analysis and graphical representation. Quantitative variables were analyzed by one-way analysis of variance (ANOVA) (with Tukey’s *post hoc* multiple-comparison test) by pairing data obtained from the experimental replicates (i.e., matched observations) and/or Student’s *t* test (two-tailed, paired), as appropriate. A *P* value of <0.05 was considered statistically significant. Statistical analysis of the distribution of the CI values was performed using the Mann-Whitney U test. *P* values of <0.05 were considered statistically significant.

### Data availability.

The data generated for this study are available upon request from the corresponding author.
