# Supplementary material for: Role of Enzymatic Activity in the Biological Cost Associated with the Production of AmpC β-Lactamases in Pseudomonas aeruginosa
Source: Microbiol Spectr. 2022 Oct 10;10(5):e02700-22. doi: 10.1128/spectrum.02700-22 (PMC9604156; doi:10.1128/spectrum.02700-22)
Supplement: Supplemental file 1 — Fig. S1 and Tables S1 to S3. Download spectrum.02700-22-s0001.pdf, PDF file, 1.3 MB [file spectrum.02700-22-s0001.pdf]

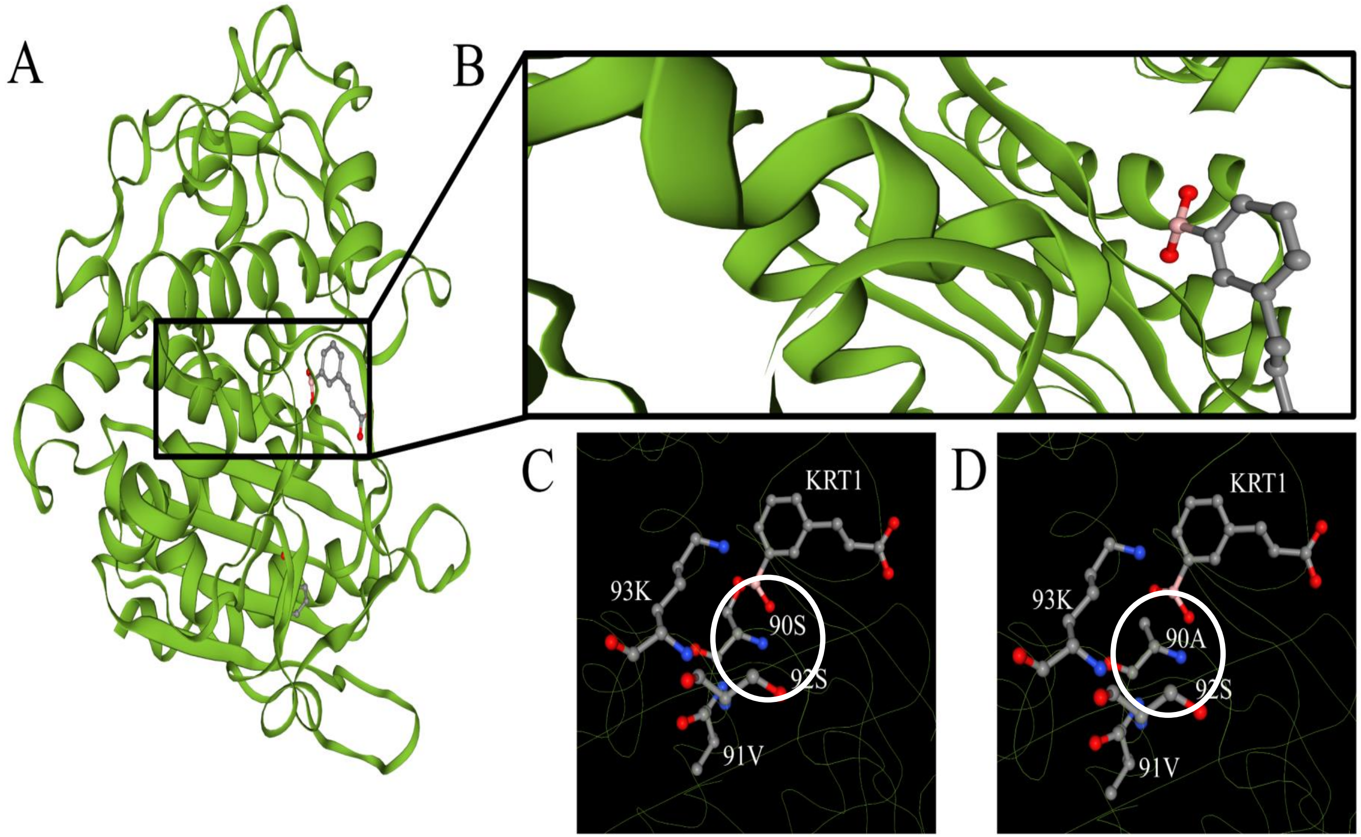

Supplementary Figure 1. An in-silico model of both the native and the disabled AmpC were created using the Expasy Swiss-model (https://swissmodel.expasy.org/) and compared through the Compare tool offered by the website, which provided the consensus sequence in green. A template containing the ligand KRT1 [3-(2-carboxyvinyl)phenyl]boronic acid] inhibitor was used to construct the model as reference.

A) General vision of the reconstructed model of both AmpCs superposed; **B)** Detail of the loop containing the active centre with the KRT1 ligand; **C)** The native and **D)** the inactivated active centre were recreated by generating individual snapshots selecting each of the positions and superposing them using the KRT1 ligand as reference. The white circle points out how the catalytic serine in position 90 interacts with the ligand whereas the structural alanine in the disabled AmpC does not.

TABLE S1. Strains and plasmids used in this work and their relevant characteristics

Strain or plasmid	Genotype/relevant characteristic(s)	Reference or source
P. aeruginosa		
PAO1	Completely sequenced reference strain	1
PAΔAC	PAO1 Δ*ampC*::lox; KO mutant in the intrinsic β-lactamase *ampC* gene	2
PAO1AC*	Wild-type *ampC* replaced by a variant with the nucleotide change T268G (Ser90Ala) disabling its enzymatic activity	This work
PAΔAG[1]	PAO1 Δ*ampG*::lox; *ampG* encodes the specific permease enabling the entry and recycling of most peptidoglycan soluble fragments into the cytosol	3
PAΔDDh2Dh3[1]	PAO1 Δ*ampD*::lox- Δ*ampDh2*::lox- Δ*ampDh3*::lox; Triple amidase-defective mutant with impaired peptidoglycan recycling and derepressed AmpC production	4
PAΔDDh2Dh3ΔAC	PAO1 Δ*ampD*::lox- Δ*ampDh2*::lox- Δ*ampDh3*::lox-Δ*ampC*::lox	2
PAΔDDh2Dh3AC*	Δ*ampD*::lox- Δ*ampDh2*::lox- Δ*ampDh3*::lox-*ampC* (T268G) (Ser90Ala)	This work
PA14	Completely sequenced reference strain,	5

	considered highly virulent and cytotoxic	
PA14AC*	Wild-type *ampC* substituted by a variant with the nucleotide change T268G (Ser90Ala) disabling its enzymatic activity	This work
PA14ΔAG	PA14 Δ*ampG*::lox	6
PA14ΔDDh2Dh3	PA14 Δ*ampD*::lox- Δ*ampDh2*::lox- Δ*ampDh3*::lox	6
PA14ΔDDh2Dh3ΔAC	PA14 Δ*ampD*::lox- Δ*ampDh2*::lox- Δ*ampDh3*::lox- Δ*ampC*::lox	6
PA14ΔDDh2Dh3AC*	Δ*ampD*::lox- Δ*ampDh2*::lox- Δ*ampDh3*::lox-*ampC* (T268G) (Ser90Ala)	This work
HUIGC-PA1	Clinical strain harboring *bla*FOX-4 β-lactamase Used as a reference for real time RT-PCR of *bla*FOX-4-like genes	7
E. coli		
XL1-Blue	Laboratory strain used for cloning experiments	8
HGURS42015	Clinical strain harboring *bla*FOX-8 β-lactamase Used as a template for cloning	9
**Plasmids**		
pUCP24	Gm^r; pUC18-based *Escherichia-Pseudomonas* multicopy shuttle vector	10
pUCPAC	Gm^r; pUCP24 containing PAO1 *ampC* wild-type gene	11
pUCPAC*	Gm^r; pUCP24 containing a PAO1 *ampC* variant	This work

	with the nucleotide change T268G (Ser90Ala) disabling its enzymatic activity	
pUCPAC T96I	Gm^r; pUCP24 containing the *ampC* variant enabling the amino-acid substitution Thr96Ile (PDC-222)	12
pUCPAC G183D	Gm^r; pUCP24 containing the *ampC* variant enabling the amino-acid substitution Gly183Asp (PDC-322)	11
pUCPAC G229-E247	Gm^r; pUCP24 containing the *ampC* variant with the encoded deletion Gly229-Glu247 (PDC-223)	12
pUCP-FOX-4	Gm^r; pUCP24 containing the *bla*FOX-4 gene	7
pUCP-FOX-8	Gm^r; pUCP24 containing the *bla*FOX-8 gene	This work

[1] When indicated for the competition experiments, the mutants in which the gentamicin resistance cassette was still present (PAΔAG::Gm and PAO1ΔDDh2Dh3::Gm) were used to enable selection of competing strains.

TABLE S2. Primers used for the analysis of gene expression in this work

Primer	Sequence (5′-3′)	PCR product size (bp)	Reference or source
RpsL-1	GCTGCAAAACTGCCCGCAACG	250	13
RpsL-2	ACCCGAGGTGTCCAGCGAACC		
[a]AC-RNA-F	GGGCTGGCCTCGAAAGAGGAC	246	4
AC-RNA-R	GCACCGAGTCGGGGAACTGCA		
[b]FOX-8-RNA-F	ATGGCCGAGCTTGCCACCT	251	This work
FOX-8-RNA-R	ATATAGGTGTGGTGCAAACCC		

[a]Since the hybridization site for these primers is conserved for the different *ampC* variants studied herein (AmpC*, T96I, N179S, G183D, and ΔG229-E247), this same pair was used to quantify the expression of all of them. [b]Since the hybridization site for these primers is conserved for *bla*FOX-4 and *bla*FOX-8, this same pair was used to quantify the expression of both β-lactamase genes.

TABLE S3. Primers used to disable the AmpC enzymatic activity

**Primer**	**Sequence (5′-3′)**	**PCR product size (bp)**	**Reference or source**
AC*1F_SacI	CGC***GAGCTC***GCGGTTGTTGTGGGTGGACAGG	800	This work
AC*2R	TCTTGCTCACCG**c**GCCGATCTCGAA		
AC*3F	ATCGGC**g**CGGTGAGCAAGACCTT	1405	This work
AC*4R_BamHI	CGC***GGATCC***GCGCCAAGGATTCAGGCGGACC		

Restriction sites are shown in bold, whereas the point mutation to enable the Ser90Ala replacement are marked in bold lowercase.

References

1. Stover CK, Pham XQ, Erwin AL, Mizoguchi SD, Warrener P, Hickey MJ, Brinkman FS, Hufnagle WO, Kowalik DJ, Lagrou M, Garber RL, Goltry L, Tolentino E, Westbrock-Wadman S, Yuan Y, Brody LL, Coulter SN, Folger KR, Kas A, Larbig K, Lim R, Smith K, Spencer D, Wong GK, Wu Z, Paulsen IT, Reizer J, Saier MH, Hancock RE, Lory S, Olson MV. 2000. Complete genome sequence of *Pseudomonas aeruginosa* PAO1, an opportunistic pathogen. Nature 406:959–964. https://doi.org/10.1038/35023079.
2. Moya B, Juan C, Alberti S, Perez JL, Oliver A. 2008. Benefit of having multiple *ampD* genes for acquiring β-lactam resistance without losing fitness and

virulence in *Pseudomonas aeruginosa*. Antimicrob Agents Chemother 52:3694–3700. https://doi.org/10.1128/AAC.00172-08.

3. Zamorano L, Reeve TM, Juan C, Moyá B, Cabot G, Vocadlo DJ, Mark BL, Oliver A. 2011. AmpG inactivation restores susceptibility of pan-beta-lactam-resistant *Pseudomonas aeruginosa* clinical strains. Antimicrob Agents Chemother 55:1990-1996. https://doi.org/10.1128/AAC.01688-10.
4. Juan C, Moyá B, Pérez JL, Oliver A. 2006. Stepwise upregulation of the *Pseudomonas aeruginosa* chromosomal cephalosporinase conferring high-level beta-lactam resistance involves three AmpD homologues. Antimicrob Agents Chemother 50:1780-1787. https://doi.org/10.1128/AAC.50.5.1780-1787.2006.
5. Lee DG, Urbach JM, Wu G, Liberati NT, Feinbaum RL, Miyata S, Diggins LT, He J, Saucier M, Déziel E, Friedman L, Li L, Grills G, Montgomery K, Kucherlapati R, Rahme LG, Ausubel FM. 2006. Genomic analysis reveals that *Pseudomonas aeruginosa* virulence is combinatorial. Genome Biol 7: R90. https://doi.org/10.1186/gb-2006-7-10-r90.
6. Pérez-Gallego M, Torrens G, Castillo-Vera J, Moya B, Zamorano L, Cabot G, Hultenby K, Albertí S, Mellroth P, Henriques-Normark B, Normark S, Oliver A, Juan C. 2016. Impact of AmpC Derepression on Fitness and Virulence: the Mechanism or the Pathway? mBio 7:e01783-16. https://doi.org/10.1128/mBio.01783-16.
7. Fraile-Ribot PA, Del Rosario-Quintana C, López-Causapé C, Gomis-Font MA, Ojeda-Vargas M, Oliver A. 2019. Emergence of Resistance to Novel β-Lactam-β-Lactamase Inhibitor Combinations Due to Horizontally Acquired AmpC (FOX-4)

in *Pseudomonas aeruginosa* Sequence Type 308. Antimicrob Agents Chemother 64:e02112-19. https://doi.org/10.1128/AAC.02112-19.

8. Bullock WO, Fernandez JM, Short JM. 1987. XL1-Blue—a high-efficiency plasmid transforming *recA Escherichia coli* strain with β-galactosidase selection. Biotechniques 5:376-379.
9. Pérez-Llarena FJ, Kerff F, Zamorano L, Fernández MC, Nuñez ML, Miró E, Oliver A, Navarro F, Bou G. 2013. Characterization of the new AmpC β-lactamase FOX-8 reveals a single mutation, Phe313Leu, located in the R2 loop that affects ceftazidime hydrolysis. Antimicrob Agents Chemother 57:5158-5161. https://doi.org/10.1128/AAC.00818-13.
10. West SE, Schweizer HP, Dall C, Sample AK, Runyen-Janecky LJ. 1994. Construction of improved *Escherichia-Pseudomonas* shuttle vectors derived from pUC18/19 and sequence of the region required for their replication in *Pseudomonas aeruginosa*. Gene 148:81-86. https://doi.org/10.1016/0378-1119(94)90237-2.
11. Cabot G, Bruchmann S, Mulet X, Zamorano L, Moyà B, Juan C, Haussler S, Oliver A. 2014. *Pseudomonas aeruginosa* ceftolozane-tazobactam resistance development requires multiple mutations leading to overexpression and structural modification of AmpC. Antimicrob Agents Chemother 58:3091-3099. https://doi.org/10.1128/AAC.02462-13.
12. Fraile-Ribot PA, Cabot G, Mulet X, Periañez L, Martín-Pena ML, Juan C, Pérez JL, Oliver A. 2018. Mechanisms leading to in vivo ceftolozane/tazobactam resistance development during the treatment of infections caused by MDR

Pseudomonas aeruginosa. J Antimicrob Chemother 73:658-663. https://doi.org/10.1093/jac/dkx424.

13. Oh H, Stenhoff J, Jalal S, Wretlind B. 2003. Role of efflux pumps and mutations in genes for topoisomerases II and IV in fluoroquinolone-resistant *Pseudomonas aeruginosa* strains. Microb Drug Resist 9:323-328. https://doi.org/10.1089/107662903322762743.
